# Comment on *Photosystem II: light-dependent oscillation of ligand composition at its active site*: existence of O6 in S_3_-state photosystem II revealed by omit maps

**DOI:** 10.1107/S2059798326003621

**Published:** 2026-05-11

**Authors:** Hongjie Li, Michihiro Suga, Jian-Ren Shen

**Affiliations:** a Center for Transformative Science, School of Life Science and Technology, and Shanghai Clinical Research and Trial Center ShanghaiTech University Shanghai201210 People’s Republic of China; b Research Institute for Interdisciplinary Science, Advanced Research Field, and Graduate School of Environmental, Life, Natural Science and Technology Okayama University Okayama700-8530 Japan; University of Cambridge, United Kingdom

**Keywords:** Kok cycle, photosystem II, oxygen evolution, water-splitting reaction, polder omit maps, XFELs, water oxidation, S-state transition, O6 insertion, pump–probe XFEL experiments

## Abstract

Photosystem II (PSII) catalyzes the light-driven oxidation of water to molecular oxygen through a series of redox states (S_0_–S_4_) in the Kok cycle. This reaction is catalyzed by a Mn_4_CaO_5_ cluster bound to PSII in the dark-stable S_1_ state, and insertion of a sixth oxygen ligand (O6) in the S_3_ state has been shown to be a key step in O—O bond formation by multiple pump–probe X-ray free-electron laser (XFEL) studies. However, a recent reanalysis of the experimental XFEL data by Wang [(2024 ▸), *Acta Cryst.* D**80**, 850–861] challenged the existence of O6, attributing the observed difference electron density to an artifact arising from a two-conformer model of O5 instead of the insertion of a new oxygen O6. Here, we reanalyzed our 2F dataset using improved omit-map strategies, including conformation-specific analyses, and clearly resolved electron density corresponding to a partially occupied O6 site. Moreover, omit maps from time-resolved serial femtosecond X-ray crystallography datasets at room temperature revealed a transient intermediate (O6*) near the Ca site shortly after the second flash, which subsequently migrated to the O6 position. These findings provide structural evidence that supports the assignment of O6 as a state-specific ligand in the S_3_ state, and are inconsistent with the interpretation proposed by Wang.

## Abstract 2

We reanalyzed our XFEL data, the results of which showed the insertion of O6 during the S_2_–S_3_ transition in water oxidation by photosystem II. These results contradict the interpretation proposed by Wang that there is no insertion of O6 during the S_2_–S_3_ transition.

## 1. Introduction

Photosystem II (PSII) is the only known enzyme in nature that catalyzes the light-driven oxidation of water to molecular oxygen, converting light energy into chemical energy. At its catalytic core is a Mn_4_CaO_5_ cluster, known as the oxygen-evolving complex (OEC), which sequentially extracts electrons from two water molecules to produce O_2_, protons and electrons; the electrons are then fed into the electron-transport chain. This catalytic process follows the Kok cycle, which divides the OEC into five discrete oxidation states termed S_*i*_ states (S_0_ to S_4_; Kok *et al.*, 1970 ▸). In the absence of light, PSII rests in a stable S_1_ state. Upon successive single-turnover flash illuminations, the system advances by one S state per flash, completing a full cycle after four flashes (Kok *et al.*, 1970 ▸). Accordingly, in flash-induced experiments, samples are commonly denoted by the number of flashes applied (*n*F), where 0F, 1F, 2F and 3F correspond to the dominant S_1_, S_2_, S_3_ and S_0_ states, respectively. Molecular oxygen is released during the S_3_→(S_4_)→S_0_ transition, with S_4_ being a transitional, short-lived intermediate (Shen, 2015 ▸; Vinyard & Brudvig, 2017 ▸; Cox *et al.*, 2020 ▸).

The crystal structure of the dark-adapted S_1_ state was obtained at 1.9 Å resolution using synchrotron X-ray radiation in 2011, which revealed the detailed geometry of the Mn_4_CaO_5_ cluster (Umena *et al.*, 2011 ▸). The OEC adopts a distorted-chair configuration, with each metal ion coordinated by bridging oxygens, amino-acid residues and water molecules (Umena *et al.*, 2011 ▸; Suga *et al.*, 2015 ▸). Since then, this structure has served as a reference for mechanistic studies of water oxidation.

The development of X-ray free-electron lasers (XFELs) has enabled time-resolved structural studies of PSII, allowing researchers to visualize intermediate states during the Kok cycle with unprecedented temporal resolution (Suga *et al.*, 2017 ▸, 2019 ▸, 2020 ▸; Kern *et al.*, 2018 ▸; Ibrahim *et al.*, 2020 ▸; Li *et al.*, 2021 ▸, 2024 ▸; Bhowmick *et al.*, 2023 ▸). Among these states, the S_3_ state has attracted particular interest, as it is the last state immediately before the release of molecular oxygen. In 2017, Suga and coworkers first reported the insertion of O6, a water molecule, in the S_3_ state using time-resolved serial femto­second crystallography (TR-SFX) at room temperature. This conclusion is based on an *F*_o(2F)_ − *F*_o(dark)_ isomorphous Fourier difference map obtained by subtracting the structure-factor amplitudes of 0F from those of 2F using the 0F model phase (Suga *et al.*, 2017 ▸). As this approach uses the same dark-state model phase for the light-illuminated state, it does not introduce additional model phase and thus minimizes model bias associated with a specific 2F structural model, although a limited degree of bias towards the 0F structure may remain. Therefore, only light-induced structural changes are highlighted in the resultant Fourier difference (*F*_o(after flash)_ − *F*_o(before flash)_) map. Subsequent studies, including work from the Berkeley group and Suga *et al.* (2019 ▸), analyzed difference electron-density maps derived from models in which O6 was not included, providing additional support for the presence of an extra oxygen ligand in the S_3_ state (Kern *et al.*, 2018 ▸; Suga *et al.*, 2019 ▸; Ibrahim *et al.*, 2020 ▸). Together, these results support the transient appearance of O6 (or Ox) in the S_3_ state and its potential role as one of the substrate O atoms in catalysis (Suga *et al.*, 2017 ▸, 2019 ▸; Kern *et al.*, 2018 ▸; Ibrahim *et al.*, 2020 ▸). The observation that the O6 atom is visible only after 2F illumination, but not after 1F illumination, reinforces the conclusion that a new oxygen (water molecule) is inserted during the S_2_–S_3_ transition (Suga *et al.*, 2019 ▸). In our recent TR-SFX experiments, we observed an O6* atom at an intermediate location near D1-Glu189 and the Ca^2+^ ion in a 1–200 µs time range following 2F, which enters into the O6 position 5 ms after 2F excitation (Li *et al.*, 2024 ▸). This observation reflects the structural changes that occur during the transition from the S_2_ state to the S_3_ state. The disappearance of O6-associated electron density has been reported after illumination with 3F, corresponding to O_2_ release during the S_3_→S_0_ transition (Bhowmick *et al.*, 2023 ▸). These results provide evidence consistent with O6 being one of the substrate O atoms involved in the formation of dioxygen.

The occupancy of O6 in the 2F dataset was estimated to be ∼50–70% (Kato *et al.*, 2018 ▸), likely due to limited excitation efficiency and water accessibility within densely packed PSII micro-crystals. Despite these experimental limitations, difference Fourier maps between the S_3_ and S_1_ or S_3_ and S_2_ states consistently reveal electron density at the O6 position, whereas no such density is observed in the S_2_–S_1_ difference Fourier map, indicating a reproducible signal associated with the S_3_ state. Although the presence of O6 in the S_3_ state is supported by multiple difference Fourier map analyses, the O5–O6 distance reported ranged from ∼1.9 to ∼2.4 Å (Suga *et al.*, 2019 ▸; Li *et al.*, 2024 ▸; Kern *et al.*, 2018 ▸; Ibrahim *et al.*, 2020 ▸); therefore, the chemical identity of O6 (oxyl versus hydroxyl species) remains under debate. These differences are likely due to a combination of partial occupancy of O6, differences in data quality and resolution, and varying refinement strategies, especially under conditions of weak electron densities.

In contrast, Wang argues that O6 is absent from the OEC following 2F excitation, based on a reanalysis of both our TR-SFX data and independent datasets from the Berkeley group (Wang *et al.*, 2021 ▸; Wang, 2024 ▸). Using the deposited data, Wang reinterpreted the structural models and attributed the density of O6 to model bias, as well as to assumptions from spectroscopic measurements. Electron paramagnetic resonance (EPR) measurements have indicated that all manganese ions should be in an Mn(IV) valence state after 2F, which requires six oxygen ligands (Cox *et al.*, 2014 ▸), and this was taken as evidence for O6 insertion in the X-ray crystallo­graphic analysis. Wang instead suggests a positional shift of O5 in the S_3_ state, and proposes that the atomic occupancies of the oxygen ligands in OEC are incomplete (Wang *et al.*, 2021 ▸; Wang, 2024 ▸).

As supporting evidence, Wang cites a 1.71 Å resolution cryo-electron microscopy (cryo-EM) study analyzing a mixed population of 1F and 2F states, which reported a Mn_4_CaO_5_ structure without O6 (Hussein *et al.*, 2024 ▸; Wang, 2025 ▸). However, the original authors repeatedly caution that the cluster may have undergone beam-induced reduction, as suggested by elongated Mn–Mn and Mn–O distances (Hussein *et al.*, 2024 ▸). Moreover, given that the sample is a mixture of 1F and 2F states, and that the excitation efficiency was not quantified, fewer PSII centers likely reached the S_3_ state (Hussein *et al.*, 2024 ▸). Thus, this cryo-EM study does not necessarily rule out the presence of O6 in the S_3_ state.

**Figure 1:**
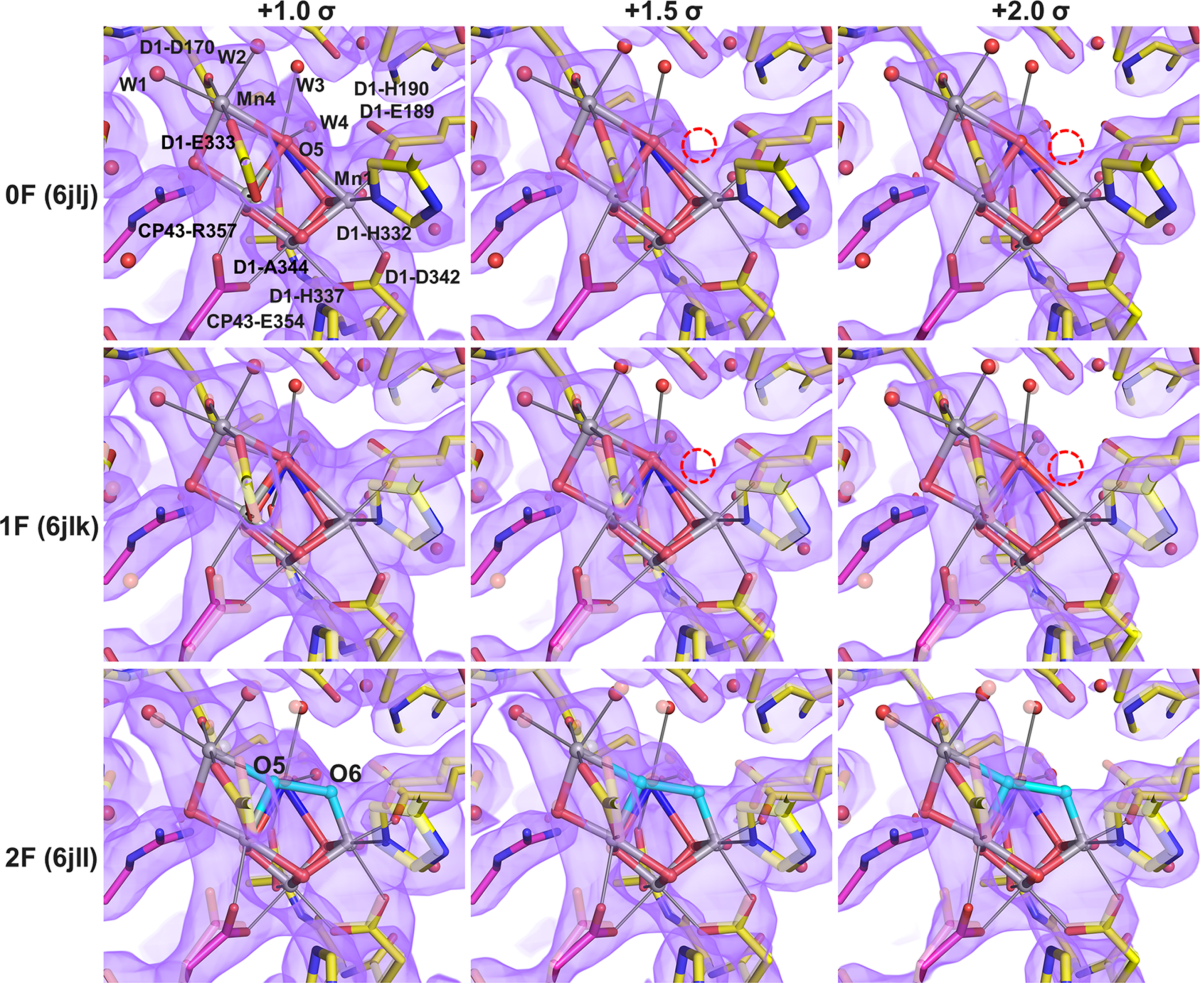
2*F*_o_ − *F*_c_ maps with the occupancies of the OEC and surrounding residues set to 0. The 2*F*_o_ − *F*_c_ maps for the 0F (PDB entry 6jlj), 1F (PDB entry 6jlk) and 2F (PDB entry 6jll) datasets are shown in magenta and contoured at 1.0σ, 1.5σ and 2.0σ, respectively, overlaid with their corresponding structural models. The S_2_-state model from the 1F dataset and the S_3_-state model from the 2F dataset are displayed as solid sticks and spheres, while the dark S_1_-state model in 1F and the S_2_-state model in 2F are shown as transparent sticks and spheres for comparison. Within the OEC, manganese ions are colored gray, calcium ions blue and O1–O5 in the S_1_ and S_2_ states are rendered in red. In the S_3_ state, O5 and O6 are represented as cyan spheres. Red dashed circles indicate the position of O6 absent in the 0F and 1F datasets.

In our previous analyses, the presence of O6 was inferred primarily from difference Fourier (*F*_o_ − *F*_o_) maps, which directly compare light-activated and reference states and are widely used to detect photoinduced structural changes while minimizing dependence on a specific structural model (Suga *et al.*, 2017 ▸, 2019 ▸; Li *et al.*, 2024 ▸). These analyses provide consistent evidence for the emergence of additional electron density that is best explained by the insertion of an oxygen (O6) near O5 in the S_3_-state component. As Wang has questioned this interpretation and argues that O6 is absent following 2F excitation (Wang *et al.*, 2021 ▸; Wang, 2024 ▸), we reassess the interpretation by Wang (2024 ▸) in two complementary ways. Firstly, we reproduce Wang’s omit-map calculation and map-processing framework on the same 2F dataset and explicitly evaluate how the inferred density near the O5–O6 region depends on *B*-factor sharpening and contour level (Figs. 1 ▸ and 2 ▸). Secondly, we apply bias-reduced omit-map analyses that account for the mixed conformational/S-state population in the 2F data (Figs. 3 ▸ and 4 ▸), and we further examine independent room-temperature time-resolved datasets that track the appearance of a transient oxygen intermediate and its subsequent migration to the O6 position following 2F (Fig. 5 ▸). Together, these analyses address a methodological bias in Wang’s interpretation and provide a consistent structural picture supporting a partially occupied O6 site associated with the S_3_ state.

## 2. Methods

### 2.1. Occupancy-based omit-map calculations for comparison with the results of Wang (2024 ▸)

**Figure 2:**
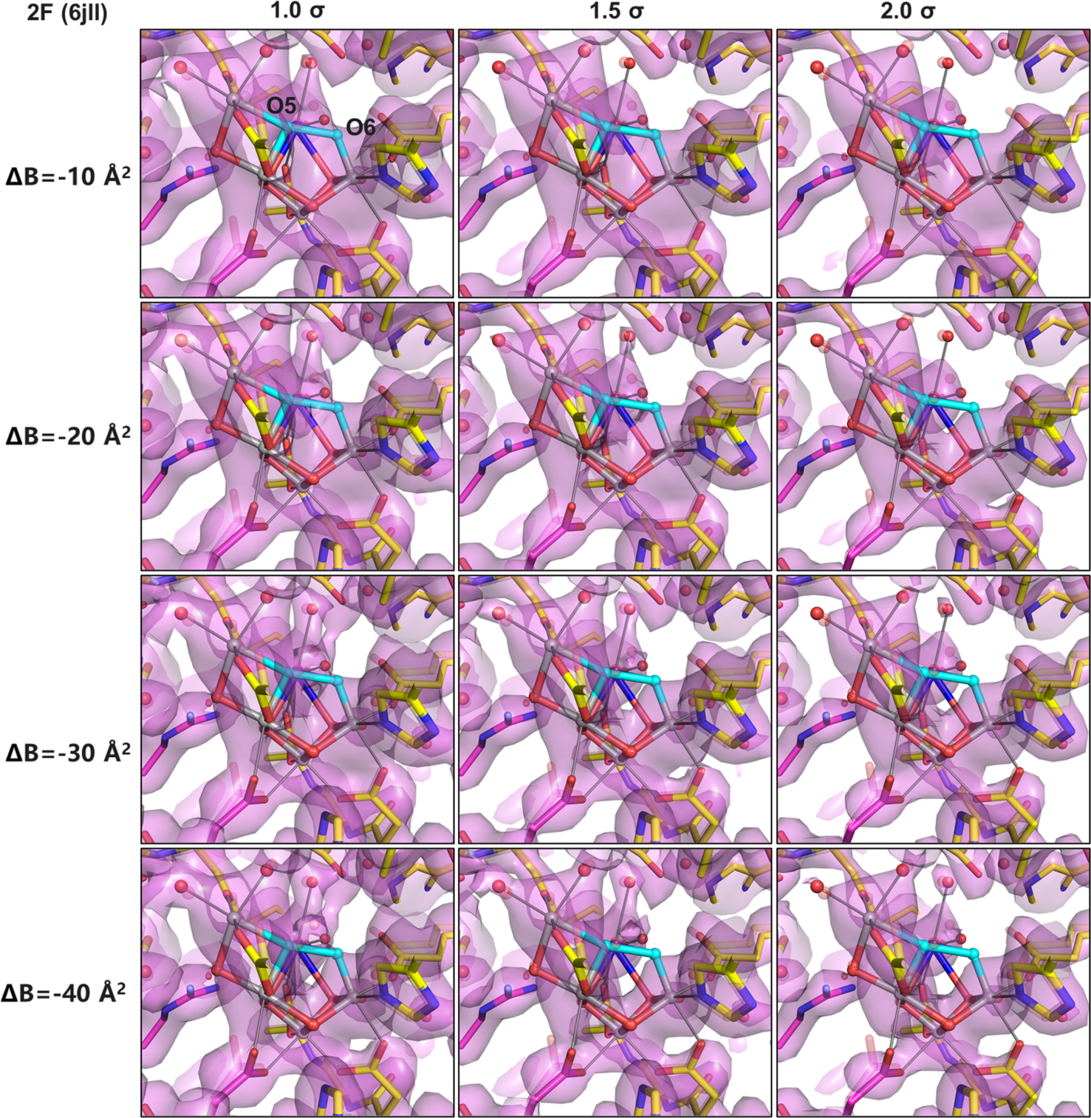
Omit maps of the OEC and its ligands calculated for the 2F dataset (PDB entry 6jll) under different map-processing conditions. The OEC and surrounding residues were omitted by setting their occupancies to zero, and all maps were calculated as 2*F*_o_ − *F*_c_ maps using *phenix.maps*. *B*-factor-sharpened maps are shown with sharpening values of −10, −20, −30 and −40 Å^2^, each contoured at 1.0σ, 1.5σ and 2.0σ, respectively. Within the OEC, manganese ions are colored gray, calcium ions blue, O1–O4 are shown in red and O5 and O6 are represented as cyan spheres.

The unsharpened 2*mF*_o_ − *DF*_c_ omit maps shown in Fig. 1 ▸ were calculated to enable a direct comparison with the results of Wang (2024 ▸), using the same occupancy-based omission strategy as employed by Wang (2024 ▸). In Wang (2024 ▸), the occupancies of selected atoms were set to zero and a zero-cycle refinement was performed using *REFMAC*5 within the *CCP*4 package (Murshudov *et al.*, 2011 ▸; Agirre *et al.*, 2023 ▸; Wang, 2024 ▸). To reproduce this omit-map calculation procedure without introducing additional refinement steps, we generated unsharpened omit maps using *phenix.maps* (*Phenix* version 1.21.2-5419; Pražnikar *et al.*, 2009 ▸; Liebschner *et al.*, 2019 ▸). For the A monomer of the 2F (PDB entry 6jll) dataset, we set the occupancies of OEC, ligated water molecules W1–W4 and the following residues to zero: D1-Asp170, D1-Glu189, D1-His332, D1-Glu333, D1-Asp342, D1-Ala344 and CP43-Glu354. This occupancy-based omission strategy removes the direct contribution of the omitted atoms to the calculated structure factors, but may retain residual model bias, as atoms with zero occupancy can still influence phase calculation and bulk-solvent treatment. Therefore, these maps are primarily used here for methodological comparison with the results of Wang (2024 ▸). The sharpened maps shown in Fig. 2 ▸ were generated from the corresponding unsharpened omit 2*mF*_o_ − *DF*_c_ maps using *phenix.auto_sharpen*, with overall isotropic *B*-factor sharpening values of −10, −20, −30 and −40 Å^2^, respectively (Terwilliger *et al.*, 2018 ▸).

### 2.2. Polder omit-map calculations

**Figure 3:**
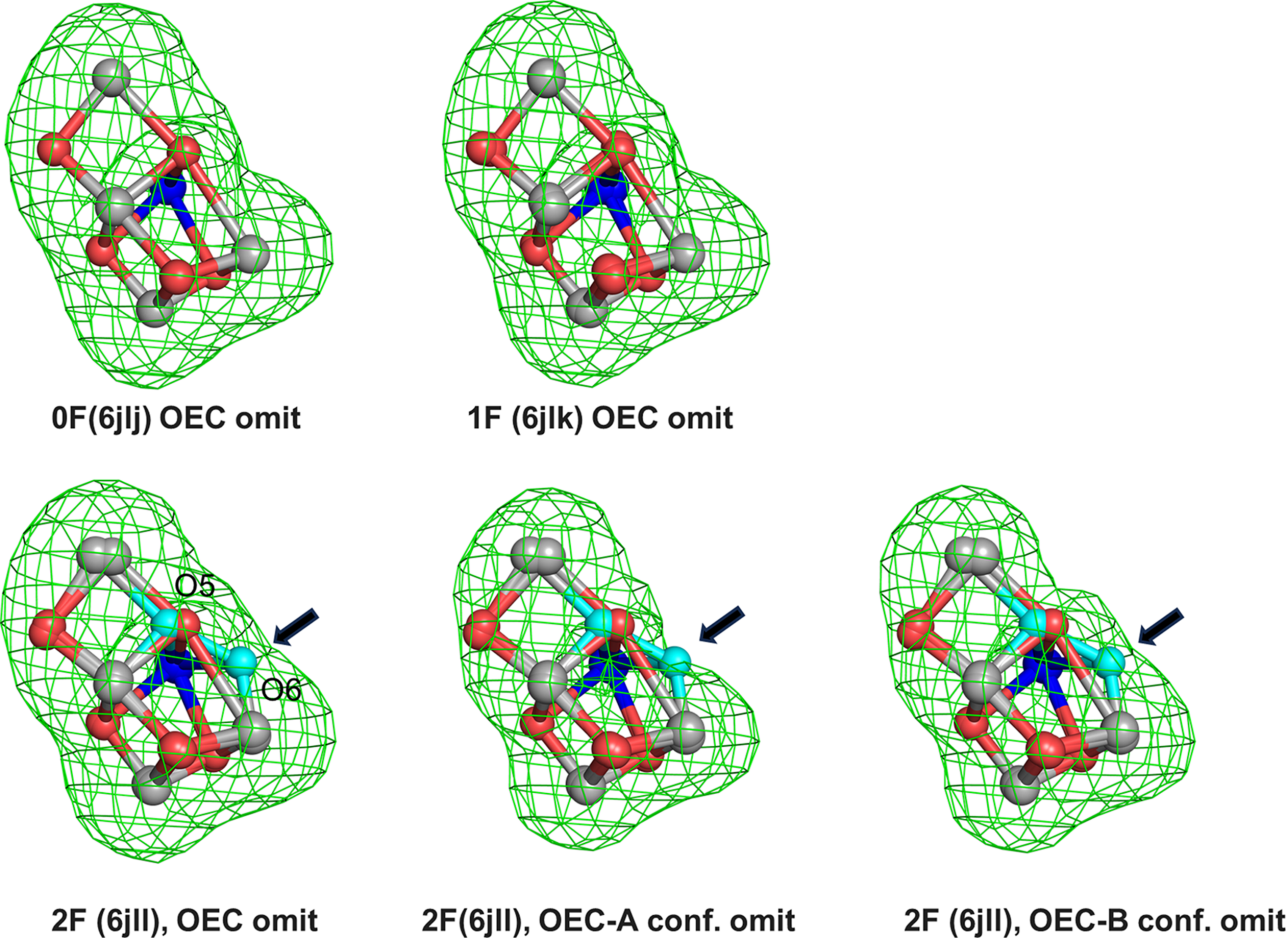
Polder omit maps of OEC. Polder omit maps of OEC in the 0F (PDB entry 6jlj), 1F (PDB entry 6jlk) and 2F (PDB entry 6jll) datasets, contoured at +4.0σ and shown in green. OEC-A conf. and OEC-B conf. in 2F indicate conformation A (S_1_/S_2_-like structure) and conformation B (S_3_-like structure), respectively. Black arrows indicate differences in the 2F omit electron density resulting from mixtures of S_1_/S_2_ and S_3_ states.

The polder omit maps shown in Figs. 3 ▸, 4 ▸ and 5 ▸ were generated using *phenix.polder* (version 1.21.2-5419; Liebschner*et al.*, 2017 ▸. 2019 ▸), which omits the target atoms and excludes bulk solvent from the omitted region, thereby allowing a more reliable visualization of weak or partially occupied electron density. Atomic coordinates and structure factors were retrieved from the PDB for the following entries: 6jlj, 6jlk, 6jll, 8irc, 8ire, 8irf, 8irh and 8iri. The high-resolution cutoffs were set to the reported resolution for each dataset, while the low-resolution limit was fixed at 20 Å to maintain consistency with the low-resolution limits used during refinement of the datasets in Suga *et al.* (2019 ▸) and Li *et al.* (2024 ▸), thereby ensuring comparable map scaling across different flash states. All other parameters were left at their default values, as recommended in the *Phenix* documentation (Liebschner *et al.*, 2019 ▸). The resulting electron-density maps were visualized using *Coot* (Emsley *et al.*, 2010 ▸) and figures were prepared using *PyMOL* (https://pymol.org/). All density maps were displayed within a 3 Å radius around the OEC.

Polder omit maps are σ-normalized difference density maps, in which σ is map-dependent and reflects statistical significance rather than electron-density values (e Å^−3^). This normalization is performed independently for each dataset and therefore does not place different maps on a common scale. As discussed by Urzhumtsev *et al.* (2014 ▸), σ values from independently normalized crystallographic maps are not suitable for quantitative comparison across datasets. Accordingly, in the present study we do not rely on σ-based contour levels for cross-dataset comparison of peak heights. Instead, we evaluate electron-density values (in e Å^−3^) at equivalent atomic positions within the OEC, extracted from polder omit maps calculated using an identical procedure for all datasets. This approach provides a quantitative and map-independent basis for comparing electron-density features across datasets.

## 3. Results

### 3.1. Evidence for the presence of O6 in the 2F state revealed by conformation-specific omit maps

In Fig. 1 of Wang (2024 ▸), omit maps of the OEC and its surrounding residues (by setting the occupancies of the specific atoms to zero) were calculated for the 2F dataset (PDB entry 6jll). Wang examined electron-density maps contoured at 1.5σ and compared unsharpened maps with a *B*-factor-sharpened map generated by applying a *B* factor of −43.5 Å^2^. Based on the appearance of a single compact density feature between Mn1 and Mn4 in the sharpened map, Wang interpreted the density as arising from a single oxygen ligand (assigned as O5), and concluded that an additional oxygen ligand is not required to explain the observed feature. However, critical examination of these maps (Fig. 1 in Wang, 2024 ▸) reveals an important issue that needs to be resolved before this conclusion can be drawn. In the original, unsharpened map there were broad electron densities in the middle between Mn1 and Mn4, which were assigned as O5 and O6 [we assigned this oxygen as originating from water molecules, as there are almost only water molecules available in the crystals (Suga *et al.*, 2017 ▸); however, we do not exclude other atoms coming into this site] in our studies and indicated by blue and red circles in row 3 of Fig. 1 of Wang (2024 ▸). However, in a *B*-factor-sharpened map the electron density is ‘sharpened’, resulting in only ‘one’ electron density in the middle between Mn1 and Mn4. This was taken as evidence for the presence of only O5 in this position. Thus, the difference appears to be between ‘unsharpened’ and ‘sharpened’ maps. We want to point out that such sharpening may disproportionately suppress low-density features of light atoms near heavy atoms, such as O6 near the Mn ions, making it unsuitable for detecting weak signals. Especially in this case, because the distance between O5 and O6 is around 2.0 Å, which is apparently too short for two independent atoms to be placed, and O5 is so close to Mn4 (also around 2.0 Å), the ‘map-sharpening’ approach may not resolve the two atoms, and thus take them as one single atom. These kinds of sharpened maps are not used in our studies (Suga *et al.*, 2017 ▸, 2019 ▸; Li *et al.*, 2021 ▸, 2024 ▸) or in the studies of the Berkeley group (Kern *et al.*, 2018 ▸; Ibrahim *et al.*, 2020 ▸; Bhowmick *et al.*, 2023 ▸).

To resolve this issue, we recalculated the 2*F*_o_ − *F*_c_ maps of the 0F, 1F and 2F data by setting the occupancies of the OEC and nearby residues to 0 (Fig. 1 ▸). At a 1.0σ contour level, the weak density at the O6 position appeared to be comparable across all three datasets, making a clear conclusion impossible. However, at higher contour levels (1.5σ and higher) the 2F map consistently exhibited stronger density than the 0F and 1F maps at the position corresponding to the O6 site (Fig. 1 ▸). Positioning of O6 in the 0F and 1F maps is impossible (indicated by red circles in Fig. 1 ▸), whereas in the 2F map O6 sits at a position covered by its densities. This observation suggests that the additional density at the O6 position is specific to the 2F state, corresponding to a weakly defined, partially occupied O6 site, in line with previous reports (Suga *et al.*, 2019 ▸).

To evaluate the robustness of Wang’s interpretation based on *B*-factor-sharpened maps, we further recalculated the omit maps for the same 2F dataset using *B*-factor-sharpening values of −10, −20, −30 and −40 Å^2^, contoured at 1.0σ, 1.5σ and 2.0σ levels (Fig. 2 ▸). For direct comparability with Wang’s approach, the occupancies of OEC and surrounding residues were set to zero in all calculations. While Wang (2024 ▸) implemented this strategy via zero-cycle refinement, we generated the corresponding 2*mF*_o_ − *DF*_c_ maps using *phenix.maps* to obtain an analogous occupancy-omitted map.

This systematic comparison shows that the visibility of electron density near the putative O6 site depends strongly on both the degree of *B*-factor sharpening and the contour level. At lower contour levels and with weaker *B*-factor sharpening, a density extending towards the O6 position is apparent, whereas increasing the sharpening strength (with a larger negative *B*-factor value) and contour threshold progressively suppresses this feature (Fig. 2 ▸). These results indicate that conclusions drawn solely from strongly sharpened (larger negative *B*-factor value) maps are sensitive to sharpening strength and contour threshold and may underestimate weak or partially occupied electron density in the O5–O6 region. One possibility is that sharpening with a stronger *B* factor (larger negative *B*-factor value) may reduce the weak densities, especially as in the intermediate state of S_3_, where O5–O6 are in intermediate positions not readily explained by normal chemistry, which then compromises the explanation. Accordingly, Fig. 2 ▸ illustrates the sensitivity of omit-map features to map-processing parameters, while a quantitative interpretation based on this figure alone is not appropriate, as described below.

The above 2*mF*_o_ − *DF*_c_ maps represent an average over a mixed population of S states and structural conformations. Previous studies have shown that the 2F dataset contains contributions from both S_1_/S_2_-like and S_3_-like states, with approximate populations of 49% and 51%, respectively (Kato *et al.*, 2018 ▸; Suga *et al.*, 2017 ▸, 2019 ▸). As a consequence, density features observed in maps calculated from the full 2F dataset cannot be directly assigned to a single S state or structural configuration.

To determine whether the electron density observed in the OEC region is conformation-specific and associated with distinct S-state geometries, we performed polder omit-map analyses in which the OEC atoms corresponding to specific conformations were omitted from the model separately. When the OEC corresponding to the S_1_/S_2_-like geometry was omitted, no significant density was observed at the O6 position in the S_1_/S_2_-like conformation (conformation A) of the 2F dataset, which is similar to those observed in the 0F and 1F datasets and consistent with the absence of O6 in this conformation of the 2F dataset (Fig. 3 ▸). In contrast, polder omit maps calculated for the S_3_-like conformation (conformation B) of the 2F dataset reveal residual electron density near the O6 position when the OEC is omitted (Fig. 3 ▸). These results indicate that the O6-associated density observed in the 2F dataset arises specifically from the S_3_-state conformation.

### 3.2. Elliptical density at the O5–O6 site is inconsistent with a simple positional shift of O5

In Fig. 2 of Wang (2024 ▸), individual omit maps were calculated for each oxygen ligand in the OEC, and it was concluded that only one oxygen ligand (O5) is present between Mn1 and Mn4. However, comparison of the omit density around O5 and O6 in the 0F, 1F and 2F datasets shows that the 2F omit map exhibits an elongated, elliptical feature bridging Mn1 and Mn4, in contrast to the compact, spherical features observed in the 0F and 1F maps. Our independent analysis of the omit maps reproduces this observation, which motivated us to examine the electron-density distribution in this region in more detail.

**Figure 4:**
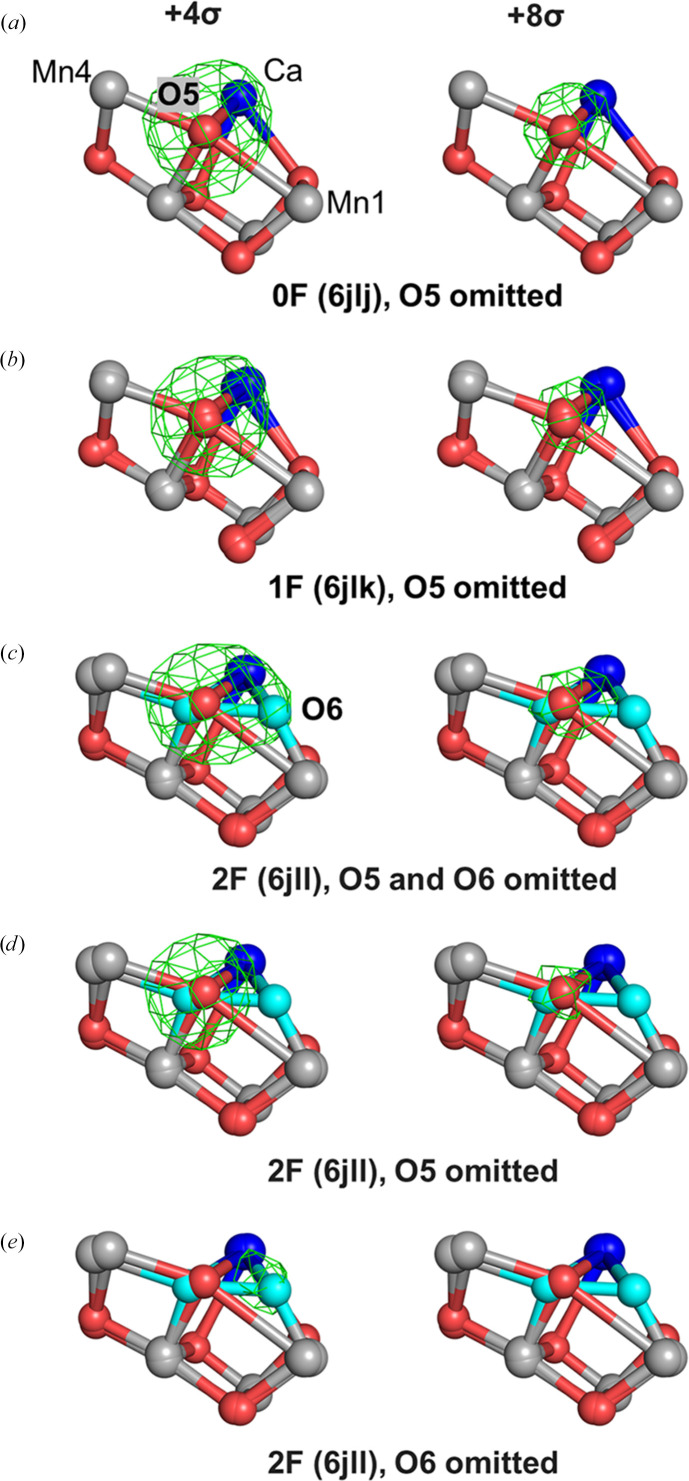
Polder omit maps of O5 and O6. (*a*, *b*, *c*) Polder omit maps with O5 omitted in 0F (PDB entry 6jlj) and 1F (PDB entry 6jlk) data, and both O5 and O6 omitted in 2F data (PDB entry 6jll). (*d*, *e*) Polder omit maps with either O5 (*d*) or O6 (*e*) omitted in the 2F dataset. All maps are contoured at +4.0σ (left) and +8.0σ (right).

We generated individual omit maps for each O atom in the 0F (PDB entry 6jlj), 1F (PDB entry 6jlk) and 2F (PDB entry 6jll) datasets. O1–O5 were omitted individually in the 0F and 1F datasets, whereas O1–O6 were omitted individually in the 2F dataset. Additionally, a combined O5/O6 omit map was calculated for the 2F dataset. Fig. 4 ▸ shows the resulting omit maps for O5 and O6 in the 0F, 1F and 2F datasets.

At a contour level of 4.0σ, the omit maps of O5 in the 0F and 1F datasets exhibit compact, spherical densities centered on O5, with no additional density extending beyond this site (Figs. 4 ▸*a* and 4 ▸*b*). This behavior is consistent with a single, well defined oxygen ligand bridging Mn1 and Mn4 in these states. In contrast, the 2F omit map with both O5 and O6 omitted reveals a distinctly different density distribution. At the same contour level (4.0σ) the density bridging Mn1 and Mn4 becomes elongated, with a prominent peak at O5 and a weaker extension towards the O6 position similar to that of the previous study (Fig. 4 ▸*c*; Suga *et al.*, 2019 ▸). This elongated feature reflects a more complex density distribution in the O5–O6 region in the 2F dataset. At higher contour levels (*e.g.* 8.0σ), density in all three maps becomes confined to the O5 region, with little to no signal at the O6 site (Figs. 4 ▸*a*–4 ▸*c*). These observations are broadly consistent with the omit maps reported by Wang (2024 ▸).

Wang proposed that the apparent O6 density at 4.0σ could be explained by positional shifts of O5, implying two alternative conformations of O5 between Mn1 and Mn4 (Wang *et al.*, 2021 ▸; Wang, 2024 ▸). To evaluate this interpretation, we directly tested whether the observed density could be accounted for by a positional shift of O5. Individually omitting O5 and O6 separately in the 2F dataset revealed well defined, spatially separated omit-map features at both sites, with the O6 feature appearing weaker than O5 (Figs. 4 ▸*d* and 4 ▸*e*). Quantitative analysis of the oxygen omit maps shows that within the same 2F dataset, the relative peak height calculated from *Coot* at the O6 position is 0.39 e^−^ Å^−3^, reaching approximately 45–70% of that observed for the other OEC O atoms including O5 (Table 1 ▸), consistent with the expected 50–70% S_3_-state population in the 2F data (Kato *et al.*, 2018 ▸). Importantly, the electron density at the original O5 position is not reduced relative to the other pre-existing oxygen ligands in the OEC (Table 1 ▸). These observations suggest that O5 does not shift towards the O6 site in the S_3_ state and are consistent with the presence of an additional, partially occupied oxygen site at the O6 position. At higher contour levels (*e.g.* 8.0σ), the density corresponding to a partially occupied oxygen site is expected to be suppressed, particularly in the presence of nearby heavy atoms. Therefore, the disappearance of O6-related density at high thresholds does not contradict its assignment as a partially occupied ligand, but is instead consistent with its limited occupancy in the 2F dataset.

Wang and coworkers also analyzed the 2F state in the Berkeley dataset (PDB entry 6w1v), refining the structure with a single OEC conformation that excluded O6 (Ibrahim *et al.*, 2020 ▸; Wang *et al.*, 2021 ▸). They attributed the residual electron density to shifts of O5 and D1-Glu189 (Wang *et al.*, 2021 ▸). Notably, this refinement yielded O5–Mn1, O5–Mn3, O5–Mn4 and O5–Ca distances of 2.63, 2.25, 2.54 and 2.60 Å, respectively, together with a positive *mF*_o_ − *F*_c_ peak of ∼3.65σ near the position corresponding to O6, which was interpreted as noise. To examine whether such residual density could indeed be accounted for by repositioning O5, we performed an analogous refinement using our independent 2F dataset (PDB entry 6jll). Specifically, a single conformation of the OEC model derived from the 0F structure (PDB entry 6jlj) was refined against the 2F data, with O6 omitted and all distance and angle restraints involving O5 removed, allowing O5 to move freely. Under these conditions, the resulting O5–metal distances (2.42–2.72 Å to Mn and Ca) remained in a similarly elongated range (Supplementary Fig. S1). Such geometries, characterized by uniformly long O5–metal distances, are difficult to reconcile with O5 acting as a stable μ-oxo or μ-hydroxo ligand bridging the OEC core, and therefore suggest a weakly bound or displaced oxygen configuration. In addition, a strong positive *mF*_o_ − *F*_c_ peak (+3.78σ) persisted near the expected O6 site (Supplementary Fig. S1), closely matching the residual density reported by Wang and coworkers. Notably, previous refinements that explicitly included O6 have shown that this residual density is not present (Suga *et al.*, 2019 ▸). Together, these observations indicate that the residual density cannot be satisfactorily explained by positional shifts of O5 alone and are best explained by the appearance of a new O atom in the S_3_ state.

### 3.3. Identification of the precursor atom O6* and its movement to O6 at room temperature

**Figure 5:**
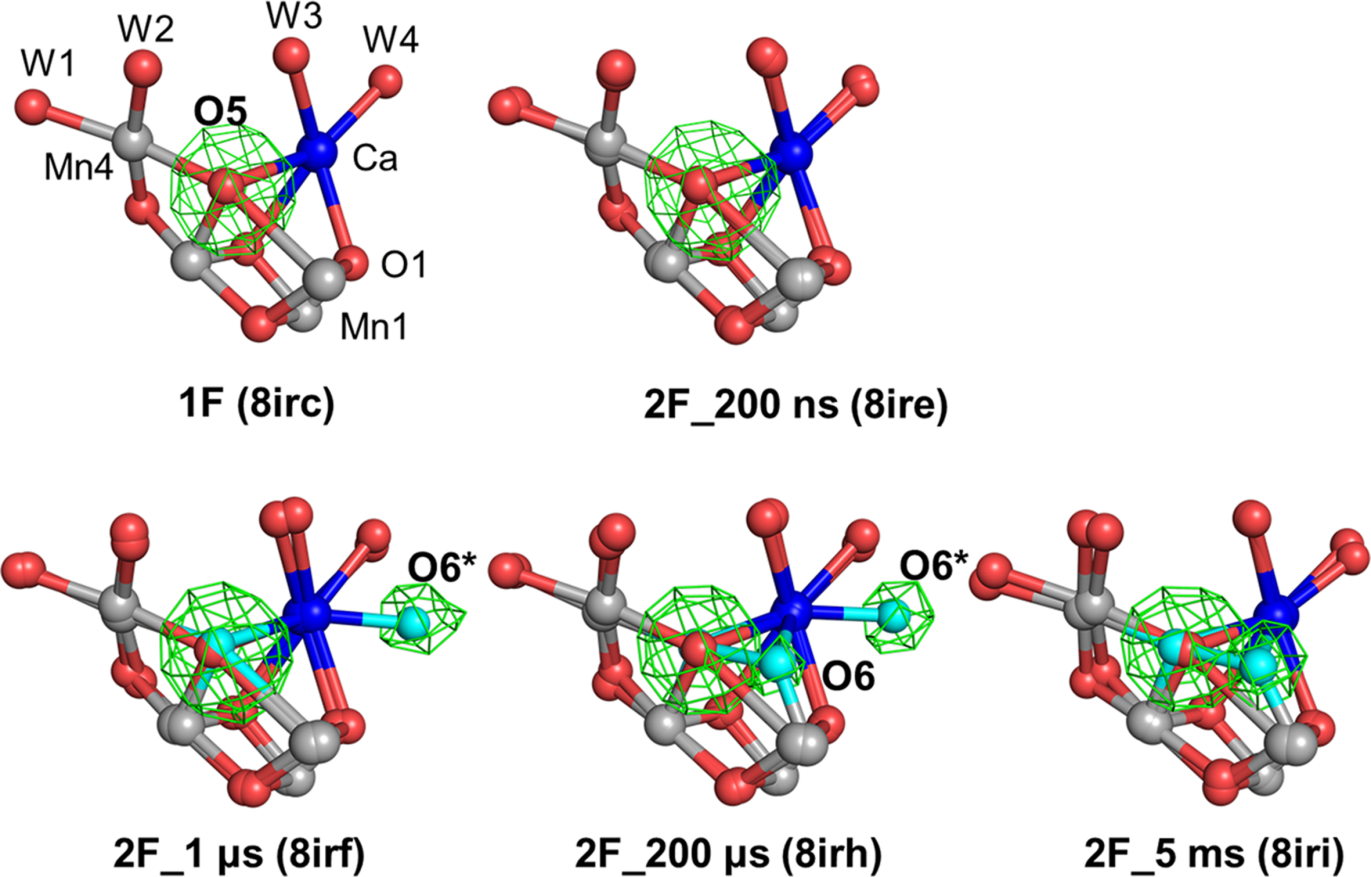
Polder omit maps of O5, O6 and O6* in data collected at room temperature. The color scheme for the OEC under different flash numbers follows that used in Figs. 3 ▸ and 4 ▸. In 1F (PDB entry 8irc) and 2F_200 ns (PDB entry 8ire), O5 is omitted. In 2F_1 µs (PDB entry 8irf), O5 and O6* are omitted independently and are displayed together. In 2F_200 µs (PDB entry 8irf), O5, O6* and O6 are omitted independently and are displayed together. In 2F_5 ms (PDB entry 8irf), O5 and O6 are omitted independently and are displayed together. In the 2F_1 µs and 2F_200 µs datasets, an intermediate oxygen position, O6*, is newly observed and is shown in cyan. The omit maps are contoured at +4.0σ.

In our recent pump–probe TR-SFX study, we employed *F*_o(after pump)_ − *F*_o(before pump)_ isomorphous difference Fourier maps to investigate the structural dynamics of OEC during the nanosecond-to-millisecond time range of the S_1_→S_2_→S_3_ transition at room temperature (Li *et al.*, 2024 ▸). This analysis revealed a transient oxygen species, designated O6*, which was initially coordinated to the Ca site between 1 and 200 µs and subsequently relocated to the O6 position between 200 µs and 5 ms following 2F. To further track this migration, we analyzed polder omit maps across the 1F_5 ms (PDB entry 8irc), 2F_200 ns (PDB entry 8ire), 2F_1 µs (PDB entry 8irf), 2F_200 µs (PDB entry 8irh) and 2F_5 ms (PDB entry 8iri) datasets. The electron density at the O5 site consistently appeared as well defined, spherical peaks with comparable intensities in all datasets (Fig. 5 ▸, Table 2 ▸), reinforcing the conclusion that O5 does not undergo positional shift during the transitions, which is inconsistent with the hypothesis that O5 contributes to the density at the O6 site. At both 1F_5 ms and 2F_200 ns no significant density is visible at the position of O6*, whereas at 2F_1 µs and 2F_200 µs O6* is visible near the Ca site, with a relative peak height of 0.27 and 0.28 e^−^ Å^−3^, respectively. By 2F_5 ms, this signal decreases to 0.18 e^−^ Å^−3^. In contrast, no density was detected at the O6 site until 1 µs following 2F, whereas a relative peak height of 0.24 e^−^ Å^−3^ emerged at the position of O6 at 200 µs and increased to 0.30 e^−^ Å^−3^ by 5 ms after 2F, suggesting the progressive incorporation of O6 into its final binding site. Together, these findings provide direct structural evidence that O6 appears only after the second flash and migrates into the OEC through a transient, calcium-associated intermediate position O6*.

### 3.4. *B*-factor variability does not provide a reliable indicator for the dioxygen source

Wang (2024 ▸) reported that several O atoms in the OEC exhibit elevated *B* factors across various PDB structures, including O3 in the A monomer of PDB entry 6jlj (0F), O3 in the B monomer of PDB entry 4ub8 (0F) and both O1 and O3 in both monomers of PDB entry 6jlp (3F) (Suga *et al.*, 2015 ▸, 2019 ▸; Wang, 2024 ▸). Elevated *B* factors were also noted for Ca in PDB entry 4ub8 and for the B conformation of O5 in monomer A of PDB entry 6jlp (3F). Based on these observations, Wang suggested that such *B*-factor elevations may reflect partial occupancy of the oxygen ligands, and proposed O1 and O3 as a potential dioxygen source for O—O bond formation (Wang, 2024 ▸).

**Figure 6:**
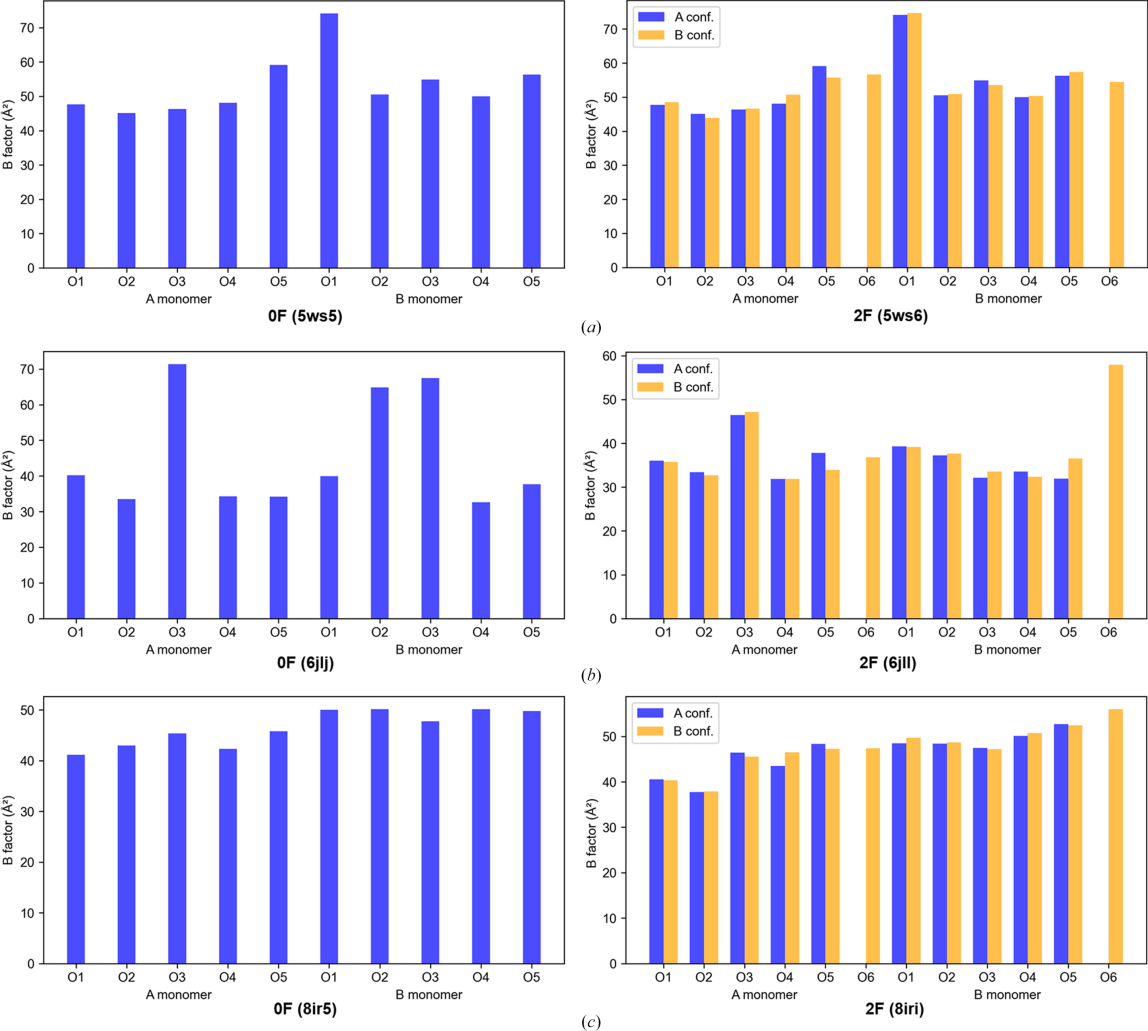
Reported *B*-factor variations of OEC O atoms in 0F and 2F datasets from models deposited in the PDB (Suga *et al.*, 2017 ▸, 2019 ▸; Li *et al.*, 2024 ▸).

To evaluate this interpretation, we examined the *B* factors of OEC O atoms in our own 0F and 2F datasets obtained at different times (Fig. 6 ▸; Suga *et al.*, 2017 ▸, 2019 ▸; Li *et al.*, 2024 ▸). Indeed, elevated *B* factors are occasionally observed, consistent with the possibility of local disorder or partial occupancy, as Wang proposed. However, no specific pattern of *B*-factor elevation is observed across the different datasets, nor is there reproducibility between the two monomers within the same PSII dimer. For example, in 0F (PDB entry 5ws5) and 2F (PDB entry 5ws6), O1, but not O3, shows a higher *B* factor in the B monomer, whereas no such elevation is observed in the A monomer. In 0F (PDB entry 6jlj) O3 exhibits elevated *B* factors in both monomers, while in 2F (PDB entry 6jll) this behavior is observed only in monomer A. In contrast, no significant *B*-factor elevation is detected for any O atom in either 0F (PDB entry 8ir5) or 2F (PDB entry 8iri) (Fig. 6 ▸). These discrepancies, both across datasets and between monomers of the same dataset, suggest that *B*-factor variability among OEC O atoms is not a robust or generalizable indicator of functional relevance in water oxidation. Such *B*-factor variations could arise from multiple sources, including local dynamics, refinement strategies, coordinate uncertainties or the conditions under which the structure was determined. Therefore, attributing mechanistic significance to individual O atoms based on *B*-factor differences solely from isolated datasets is insufficient and may lead to overinterpretation. We further note that Wang inferred fluctuations in the number of O atoms present in the dark-stable S_1_ state based on *B*-factor and occupancy analyses. However, elevated *B* factors or refined occupancies below unity do not imply the absence of an atom. In all reported crystallographic datasets, the refined occupancies of the five O atoms forming the Mn_4_CaO_5_ core remain well above 0.5, supporting the presence of a complete Mn_4_CaO_5_ cluster in the S_1_ state. Interpreting relative *B*-factor differences as evidence for the absence of specific O atoms is therefore not justified.

Elucidation of the water-splitting mechanism demands an integrative approach that combines structural analysis with spectroscopy and computational modeling. In this context, the presence of O6 is supported by multiple independent datasets and complementary experimental approaches, including *F*_o(after pump)_ − *F*_o(before pump)_ difference maps, omit-map analyses and time-resolved measurements, as well as by independent studies from our group (Suga *et al.*, 2017 ▸, 2019 ▸; Li *et al.*, 2024 ▸), the Berkeley group (Kern *et al.*, 2018 ▸; Ibrahim *et al.*, 2020 ▸; Bhowmick *et al.*, 2023 ▸) and corroborating spectroscopic data (Cox *et al.*, 2014 ▸). This convergence of evidence provides robust structural support for the assignment of O6 as a water-derived O atom involved in O—O bond formation.

## 4. Discussion

The mechanism of the water-splitting reaction has long been a central question in photosynthetic research. Earlier studies by the Yano group did not resolve O6 in the S_3_ state, but these datasets were obtained at lower resolutions or characterized by a lower multiplicity in the highest resolution shell, which likely limited the detectability of weak or partially occupied O6 in the S_3_ state (Young *et al.*, 2016 ▸). Subsequent higher resolution XFEL studies, including our TR-SFX analyses, as well as those by Yano and coworkers, have consistently identified the insertion of a new oxygen species, designated O6 or Ox, into the OEC following 2F (Suga *et al.*, 2017 ▸, 2019 ▸; Kern *et al.*, 2018 ▸; Ibrahim *et al.*, 2020 ▸). The consistent identification of O6 insertion provides critical structural insight into the catalytic step underlying water oxidation. However, Wang (2024 ▸) reanalyzed our 2F dataset and concluded that only O5 is present between Mn1 and Mn4, thereby challenging the existence of O6 (Suga *et al.*, 2019 ▸; Wang, 2024 ▸). To address this discrepancy, we conducted an independent re-evaluation of the same dataset and observed electron density consistent with a partially occupied O6 site in the S_3_ state.

Wang’s interpretation was primarily based on omit maps in which the occupancies of the entire OEC and its coordinating ligands were set to zero, a strategy described as ‘model-bias-free’ and independent of manganese oxidation assumptions (Wang, 2024 ▸). In the unsharpened omit maps presented in Wang (2024 ▸), electron density broadly spans the O5 and O6 regions in the S_3_ state. However, after applying *B*-factor sharpening, this density collapses into a single compact density feature located between Mn1 and Mn4, which was interpreted as evidence that only one O atom, O5, is present between these metal ions in the S_3_ state (Wang, 2024 ▸). To evaluate the robustness of this conclusion, we reanalyzed our 2F data with the same map-sharpening procedure as used in Wang (2024 ▸), but systematically varied the *B*-factor values applied during sharpening (Figs. 1 ▸ and 2 ▸). We find that at weak sharpening or in the absence of sharpening, electron density at the O6 position is clearly observable. In contrast, at stronger sharpening values (*B* factors more negative than −30 Å^2^), the density feature of O6 becomes weaker and eventually disappears. Because Wang (2024 ▸) applied a sharpening value of −43.5 Å^2^, the absence of the O6 density in his sharpened maps can be attributed to the choice of sharpening parameters rather than to the true absence of O6. These observations demonstrate that the conclusions drawn from sharpened maps are highly sensitive to the *B*-factor values used. *B*-factor sharpening preferentially enhances intense features associated with nearby heavy atoms such as manganese and calcium, while simultaneously suppressing weaker and more diffuse density arising from partially occupied light atoms. As a consequence, weak structural features such as O6, located in close proximity to strong metal-associated densities, may become obscured under strong sharpening conditions.

An additional consideration is that the proposed O5–O6 separation in the intermediate S_3_ state is around 2.0 Å. At this distance, aggressive map sharpening may result in the merging of closely spaced density features into a single apparent peak, rather than resolving them as two distinct O atoms. This further limits the ability of strongly sharpened maps to reliably assess the presence of an additional oxygen ligand in the intermediate structure of OEC.

Considering that the omit 2*mF*_o_ − *DF*_c_ map of the full OEC and surrounding residues can obscure weak or partially occupied features such as O6, especially in the presence of structural heterogeneity within the 2F dataset, we performed conformation-specific polder omit-map analyses to disentangle overlapping density contributions (Fig. 3 ▸). In this analysis, the two coexisting conformations in the 2F dataset were treated separately: conformation A, which resembles the S_1_/S_2_ states and does not include O6, and conformation B, which corresponds to the S_3_ state and contains the O6 atom (Fig. 3 ▸). When analyzed in this manner, electron density at the O6 position is observed only in conformation B, whereas no corresponding density is detected in conformation A. This conformation-specific behavior indicates that O6-associated density arises from the S_3_-state conformation of the 2F dataset, rather than from averaging effects or positional disorder in the S_1_/S_2_-like population. By resolving these two conformations in the mixed S-state populations inherently present in the 2F dataset, this approach minimizes averaging artifacts present in global omit maps and allows weak, state-specific features to be evaluated more reliably. The results therefore provide structurally consistent support for the presence of O6 as a ligand associated specifically with the S_3_ state.

Wang further proposed that the electron density observed near the O6 position could be explained by a positional shift of O5, rather than the incorporation of a distinct additional oxygen ligand (Wang *et al.*, 2021 ▸; Wang, 2024 ▸). This interpretation represents a plausible alternative and therefore warrants careful evaluation in light of the available structural evidence. If the observed density were attributable to a positional shift of O5, several conditions would be expected to hold simultaneously. Firstly, the electron density at the original O5 position should be reduced relative to other pre-existing OEC O atoms or the original O5 atom. Secondly, density features near the proposed O6 position should not be resolvable as a distinct site when O5 is omitted. Thirdly, refinement models that exclude O6 should yield chemically reasonable coordination geometries for O5. Our results do not satisfy these expectations. Omit-map analyses show that when O5 and O6 are omitted individually in the 2F dataset, well defined and spatially separated electron-density peaks are observed at both positions (Fig. 4 ▸). The intensity of the O6-associated peak reaches approximately 45–70% of that observed for O5 and other OEC O atoms (Table 1 ▸), consistent with a partially occupied ligand rather than with density smearing arising from positional disorder of O5. Importantly, omission of O5 does not lead to a reduction in the electron density at the original O5 site relative to other oxygen ligands (Table 1 ▸), which is inconsistent with a simple positional shift model.

Furthermore, refinement attempts that exclude O6 and allow O5 to move freely result in uniformly elongated Mn–O5 and Ca–O5 distances, together with persistent positive difference density near the O6 position (Supplementary Fig. S1). Such geometries are difficult to reconcile with O5 functioning as a stable μ-oxo or μ-hydroxo ligand bridging the OEC core. In contrast, inclusion of O6 in the model restores chemically reasonable coordination distances and eliminates the residual difference density. Taken together, these observations indicate that the positional shift model for O5 does not provide a self-consistent explanation of the electron-density distribution, coordination geometry and occupancy behavior observed in the 2F dataset. Instead, the data are more naturally explained by the presence of an additional, partially occupied oxygen ligand at the O6 position associated with the S_3_ state.

Beyond the static analyses, our time-resolved pump–probe TR-SFX experiment provides direct evidence for the dynamic incorporation of O6 into the OEC (Fig. 5 ▸; Li *et al.*, 2024 ▸). In this measurement, a transient oxygen species, designated O6*, was initially detected near the Ca site between 1 and 200 µs after 2F, which gradually migrates to the canonical O6 position by 5 ms. This temporally resolved progression, from the appearance of O6* to its disappearance and the appearance of O6, not only delineates a plausible ligand-delivery pathway, but also argues against interpretations attributing O6 density to static disorder of O5 or modeling artifacts. Notably, the electron density at O5 remained largely unchanged throughout this period (Table 2 ▸), arguing against positional displacement of O5 as a source of the observed signal. We should point out that the O6 density is observed only after 2F, but does not appear in 0F or 1F samples, consistent with O6 insertion during the S_2_ to S_3_ transition. Independent time-resolved TR-SFX studies by Yano and coworkers reported the disappearance of O6 following 3F (Bhowmick *et al.*, 2023 ▸), supporting its role as a transient intermediate specific to the S_3_ state. Taken together, these findings capture O6 at two key stages of the Kok cycle, its incorporation after 2F and its disappearance after 3F, reinforcing its functional relevance and dynamic behavior during water oxidation.

Wang also argued that elevated *B* factors may identify catalytically relevant O atoms and proposed O1 or O3 as possible sources of dioxygen (Wang, 2024 ▸). However, our systematic analysis across multiple datasets shows that *B*-factor variations among OEC O atoms are inconsistent across different structures and even between monomers within the same PSII dimer (Fig. 6 ▸). Such variability is more plausibly attributed to factors such as refinement strategies, resolution variations or intrinsic structural flexibility, rather than to a specific mechanistic role in water oxidation. An elevated *B* factor may reflect a partial occupancy or increased local disorder of a given O atom in a particular structure, but it does not necessarily indicate the absence of that O atom. Across all available crystallographic datasets, the Mn_4_CaO_5_ core is consistently observed in the S_1_ and S_2_ states, with the refined occupancies of all five oxygen ligands remaining well above 0.5. Thus, the crystallographic evidence does not support oscillations in the number of oxygen ligands within the Mn_4_CaO_5_ cluster during these states, and the *B*-factor differences alone do not provide a reliable basis for assigning the source of dioxygen.

In this context, the structural identification of O6 should be viewed as a robust, experimentally grounded observation rather than an inference derived from a single analytical approach. The convergence of difference Fourier analyses, conformation-specific omit maps and time-resolved structural snapshots provides a coherent framework in which O6 emerges as a state-specific ligand of OEC in the S_3_ state. Importantly, these conclusions are drawn directly from crystallographic observables and do not rely on assumptions derived from spectroscopic interpretations. The remaining challenge is therefore not the existence of O6, but its precise chemical nature and its role in O—O bond formation. Discriminating between oxyl, hydroxyl or related protonation states will require coordinated advances in ultrafast structural methods, high-level spectroscopy and quantum-chemical modeling. By establishing the structural reality of O6 and its dynamic behavior during the Kok cycle, the present work provides a solid foundation upon which future mechanistic studies of photosynthetic water oxidation can be built.

## Supplementary Material

Supplementary Figure S1. DOI: 10.1107/S2059798326003621/rr5260sup1.pdf

## Figures and Tables

**Table 1:** Statistical significance (σ levels) and electron-density values (e Å^−3^, shown in parentheses) of omit-map features at the O1–O6 sites in Suga *et al.* (2019 ▸) Values are obtained from independent polder omit maps. A dash (—) indicates that electron density is not detectable at the corresponding site.

	O1	O2	O3	O4	O5	O6
0F (PDB entry 6jlj)	13.5 (0.78)	12.7 (0.73)	9.7 (0.56)	14.3 (0.82)	13.7 (0.79)	—
1F (PDB entry 6jlk)	14.9 (0.85)	12.6 (0.72)	12.6 (0.72)	13.3 (0.76)	13.5 (0.78)	—
2F (PDB entry 6jll)	14.5 (0.86)	12.3 (0.73)	10.5 (0.63)	13.5 (0.80)	12.6 (0.75)	6.6 (0.39)

**Table 2:** Statistical significance (σ levels) and electron-density values (e Å^−3^, shown in parentheses) of omit-map features at the O1–O6 and O6* sites in Li *et al.* (2024 ▸) Values are obtained from independent polder omit maps. A dash (—) indicates that electron density is not detectable at the corresponding site.

	O1	O2	O3	O4	O5	O6	O6*
1F (PDB entry 8irc)	15.7 (0.76)	15.1 (0.73)	13.6 (0.65)	13.0 (0.63)	11.0 (0.53)	—	—
2F_1 µs (PDB entry 8irf)	16.0 (0.74)	16.0 (0.74)	13.6 (0.64)	12.4 (0.57)	11.2 (0.52)	—	5.9 (0.27)
2F_200 µs (PDB entry 8irh)	16.3 (0.77)	14.8 (0.70)	13.6 (0.64)	12.6 (0.60)	10.6 (0.50)	5.0 (0.24)	5.8 (0.28)
2F_5 ms (PDB entry 8iri)	15.9 (0.76)	15.1 (0.72)	14.2 (0.68)	12.7 (0.61)	10.6 (0.51)	6.3 (0.30)	3.7 (0.18)
