# Supplementary material for: Comment on Photosystem II: light-dependent oscillation of ligand composition at its active site: existence of O6 in S3-state photosystem II revealed by omit maps
Source: Acta Crystallogr D Struct Biol. 2026 May 11;82(Pt 6):574–86. doi: 10.1107/S2059798326003621 (PMC13224924; doi:10.1107/S2059798326003621)
Supplement: Supplementary file 1 [file d-82-00574-sup1.pdf]

STRUCTURAL
BIOLOGY

Volume 82 (2026)

Supporting information for article:

Comment on *Photosystem II: light-dependent oscillation of ligand composition at its active site*: existence of O6 in S3-state photosystem II revealed by omit maps

Hongjie Li, Michihiro Suga and Jian-Ren Shen

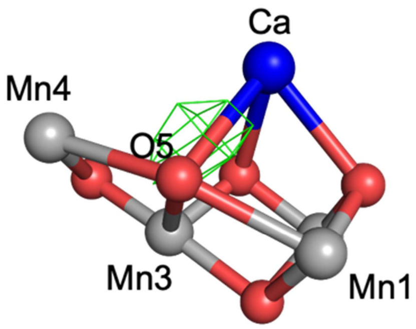

Figure S1 Model of the Mn_4CaO_5 cluster in the 2F state (6jll) refined with a single O5 atom, superimposed with the omit map omitting the putative O6 atom. In the resulting model, the O5-Mn1, O5-Mn3, O5-Mn4 and O5-Ca distances are 2.60, 2.42, 2.60, and 2.72 Å, respectively, and a positive density near the O6 atom is visible.
